# The Influence of Material Properties and Wall Thickness on Predicted Wall Stress in Ascending Aortic Aneurysms: A Finite Element Study

**DOI:** 10.1007/s13239-024-00756-9

**Published:** 2024-10-25

**Authors:** Yu Zhu, Selene Pirola, M. Yousuf Salmasi, Sumesh Sasidharan, Serena M. Fisichella, Declan P. O’Regan, James E. Moore Jr, Thanos Athanasiou, Xiao Yun Xu

**Affiliations:** 1 Department of Chemical Engineering, Imperial College London, London, UK; 2 Department of Biomechanical Engineering, Delft University of Technology, Delft, Netherlands; 3 Department of Surgery and Cancer, Imperial College London, London, UK; 4 Department of Bioengineering, Imperial College London, London, UK; 5 Politecnico di Milano, Milan, Italy; 6 MRC Laboratory of Medical Sciences, Imperial College London, London, UK

**Keywords:** Finite element analysis, Material property, Wall thickness, Ascending aortic aneurysm, Peak wall stress

## Abstract

**Purpose:**

Finite element analysis (FEA) has been used to predict wall stress in ascending thoracic aortic aneurysm (ATAA) in order to evaluate risk of dissection or rupture. Patient-specific FEA requires detailed information on ATAA geometry, loading conditions, material properties, and wall thickness. Unfortunately, measuring aortic wall thickness and mechanical properties non-invasively poses a significant challenge, necessitating the use of non-patient-specific data in most FE simulations. This study aimed to assess the impact of employing non-patient-specific material properties and wall thickness on ATAA wall stress predictions.

**Methods:**

FE simulations were performed on 13 ATAA geometries reconstructed from computed tomography angiography (CTA) images. Patient-specific material properties and wall thicknesses were made available from a previous study where uniaxial tensile testing was performed on tissue samples obtained from the same patients. The ATAA wall models were discretised with hexahedral elements and prestressed. For each ATAA model, FE simulations were conducted using patient-specific material properties and wall thicknesses, and group-mean values derived from all tissue samples included in the same experimental study. Literature-based material property and wall thickness were also obtained from the literature and applied to 4 representative cases. Additional FE simulations were performed on these 4 cases by employing group-mean and literature-based wall thicknesses.

**Results:**

FE simulations using the group-mean material property produced peak wall stresses comparable to those obtained using patient-specific material properties, with a mean deviation of 7.8%. Peak wall stresses differed by 20.8% and 18.7% in patients with exceptionally stiff or compliant walls, respectively. Comparison to results using literature-based material properties revealed larger discrepancies, ranging from 5.4% to 28.0% (mean 20.1%). Bland-Altman analysis showed significant discrepancies in areas of high wall stress, where wall stress obtained using patient-specific and literature-based properties differed by up to 674 kPa, compared to 227 kPa between patient-specific and group-mean properties. Regarding wall thickness, using the literature-based value resulted in even larger discrepancies in predicted peak stress, ranging from 24.2% to 30.0% (mean 27.3%). Again, using the group-mean wall thickness offered better predictions with a difference less than 5% in three out of four cases. While peak wall stresses were most affected by the choice of mechanical properties or wall thickness, the overall distribution of wall stress hardly changed.

**Conclusions:**

Our study demonstrated the importance of incorporating patient-specific material properties and wall thickness in FEA for risk prediction of aortic dissection or rupture. Our future efforts will focus on developing inverse methods for non-invasive determination of patient-specific wall material parameters and wall thickness.

**Supplementary Information:**

The online version contains supplementary material available at 10.1007/s13239-024-00756-9.

## Introduction

Thoracic aortic aneurysms (TAA) are a degenerative disease characterised by a permanent dilatation of the aortic wall. There are approximately 5–10 new TAA cases per 100,000 patients annually [1], with 60% occurring in the aortic root or ascending aorta [2]. As the aneurysm expands, it may rupture which can be fatal. An estimated mortality rate of 59% has been reported for patients with ruptured TAA before they reached a hospital [3]. Current international guidelines for intervention rely on measuring the maximum diameter, and surgical repair of asymptomatic aneurysms of the ascending aorta is usually recommended when the maximum diameter is ≥ 5.5 cm, while a lower threshold of 5 cm can be considered in patients with Marfan syndrome or bicuspid aortic valves [4]. However, the incidence of acute dissection or rupture was reported to be 18.3% for aneurysms < 5 cm, among 370 TAA patients [5]. Clearly, there is a pressing need for better prognostication, and this can potentially be achieved by developing a predictive model for personalised assessment of risk of rupture.

From a biomechanical perspective, the aortic wall loses its elasticity as the aorta expands, and the dilated region may experience elevated stress [6]. If the local stresses exceed the mechanical strength of the wall, rupture may occur. While wall stress is impossible to be measured directly in vivo, image-based finite element analysis (FEA) has been used extensively to calculate wall stress in ascending TAAs (ATAA) [7–11]. The accuracy of the computed stress is highly dependent on several key components involved in a FEA simulation: the aortic wall geometry including its thickness, the choice of constitutive model for wall mechanical behaviour and material properties, as well as physiological loading conditions.

Although the three-dimensional (3D) patient-specific aortic lumen geometry can be easily reconstructed using medical imaging data, segmentation of the wall and its connective components is still challenging, due to the limitations in imaging spatial resolution. As a result, a uniform wall thickness was commonly assumed in previous FEA studies, with its value being either measured ex-vivo [7, 8] or obtained from other studies [9–11]. Similar, deriving individual aortic wall material properties from non-invasively acquired in vivo data is extremely difficult and costly, especially when considering its great variability among different aortic regions [12]. Although aortic wall material properties can be determined through mechanical testing, such data may be absent due to the limited availability of ATAA tissues. As a result, averaged material properties obtained from other groups of patients have been used [13–15].

Using non-patient-specific data raises concerns about the reliability of FEA for predicting the risk of ATAA rupture individually. Therefore, in this study, our aim was to examine the impact of using non-patient-specific material properties or wall thickness on predicted wall stresses by comparing the results with those of FEA simulations based on patient-specific mechanical properties derived from uniaxial tensile testing on individual tissue samples. To the authors’ knowledge, such a detailed quantitative assessment of the errors arising from the use of non-patient-specific wall material properties and thicknesses has not been reported in the literature. In addition, Bland-Altman analyses were performed to provide a more complete quantification of discrepancies in wall stress predictions across models with varying material properties.

## Methods

### Data Acquisition

As reported in a separate study [16], a total of 354 tissue samples were cut from 34 surgically resected ATAA specimens to conduct thickness measurements and uniaxial tensile testing. The experimental procedure and results can be found in [16]. Computed tomography angiography (CTA) images acquired prior to surgery were available for 13 out of 34 patients. These images were used for 3D surface reconstruction of the ascending aorta, which was then used to create wall models for FEA. The study received ethical approval from the Health Research Authority and Regional Ethics Committee (17/NI/0160, August 2017).

### Geometry Reconstruction and Mesh Generation

Thirteen patient-specific geometries of ATAA were reconstructed from CTA images using Mimics 24.0 (Materialise, Leuven, Belgium). For each patient, only the ascending aorta was included to reduce computational time, while the involvement of aortic root depended on whether a dilatation had occurred. Subsequently, the reconstructed ATAA surfaces were imported into SPACECLIAM (ANSYS, Canonsburg, PA, United States) and offset outwardly by a uniform thickness (1.6–2.4 mm) equivalent to the ex-vivo measured thickness on tissue samples for each patient. The patients’ characteristics and the corresponding wall thickness measurements [16] are reported in Table 1.

**Table 1:** Patients characteristics and the averaged wall thickness for each patient.

Patient	Gender (M/F)	Blood pressure (mmHg)	Clinical presentations	Surgery undertaken	Averaged wall thickness (mm) [16]
C1	F	167/106	Dilated root and ascending aorta	AAR	2.2
C2	M	128/78	Dilated root and ascending aorta	ARR	2.2
C3	F	141/80	Dilated ascending aorta	AAR	2.4
C4	F	155/61	Dilated ascending aorta	AVR + AAR	2.4
C5	F	131/82	Dilated root	ARR	1.6
C6	M	138/99	Dilated root	VSRR	2.0
C7	M	140/108	Dilated ascending aorta	AVR + AAR	1.8
C8	F	137/61	Dilated ascending aorta	ARR + hemiarch replacement	2.1
S1	F	153/60	Dilated ascending aorta	AVR + AAR + FET	2.2
S2	M	124/88	Dilated ascending aorta	Bentall	2.3
S3	M	177/132	Dilated root	VSRR	2.1
S4	M	139/86	Dilated root	Bentall procedure	2.2
S5	F	115/78	Dilated ascending aorta	AAR	1.9

AVR = aortic valve replacement, AAR = ascending aorta replacement, FET = frozen elephant trunk; ARR = aortic root repair; VSRR = valve sparing aortic root replacement

ANSYS Meshing (ANSYS, Canonsburg, PA, United States) was used to generate hexahedral structural elements with hybrid formulation (C3D8H) in Abaqus. An average element size of 1.2 mm was chosen based on the mesh independent tests, where stress magnitudes predicted by a finer mesh differed < 1% compared to the adopted mesh (Supplementary Materials S1). Each model was 3 elements thick and consisted of approximately 22,584–41,302 elements and 63,190–115,084 nodes, respectively, depending on the geometric size.

### Patient-Specific and Averaged Material Properties

The stress-strain curves obtained from uniaxial tensile testing [16] were fitted using the third order Yeoh hyperelastic material model, which can be described as:

$$\:W=\:\sum\:_{1}^{3}{C}_{i}\:{({I}_{1}-3)}^{i}$$

where *W* is the strain energy density, $\:{C}_{i}$ is the material constants with $\:i$ = 1, 2, 3, for the third order model, and $\:{I}_{1}$ is the first deviatoric invariant. The Yeoh material model was selected for comparative purpose because the literature-based material properties were taken from published results using this material model [17].

**Fig. 1:**
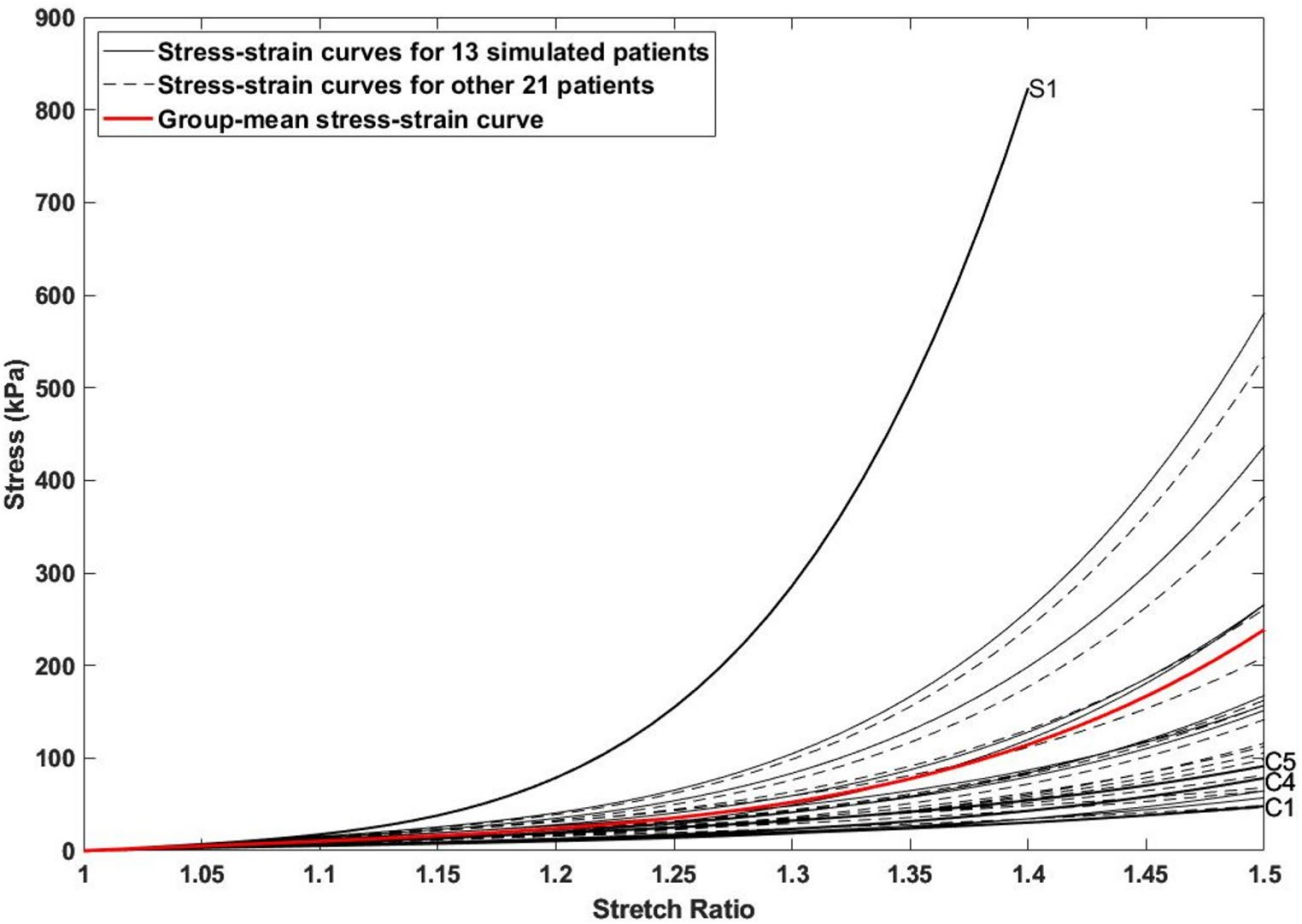
Illustration of all patient-specific stress-strain curves derived from individual tissue samples, along with a group-mean stress-strain curve (red solid curve) calculated by averaging all patient-specific data. Thirteen patients with available CTA images for FE simulations are highlighted by black solid lines, while data for the other 21 patients are shown in black dashed curves. Additional FE simulations were performed on 4 selected ATAA models: C1, C4, C5, and S1. In this context, ‘C’ represents patients with a more compliant wall material property than the group-mean value, while ‘S’ denotes patients with a stiffer wall material property

For each ATAA geometry, two sets of material properties were used for FEA simulations: patient-specific material properties obtained by averaging all tested tissue samples from each specimen (4 to 11 tissues per specimen), and the group-mean material property derived from all experimentally tested tissue samples (*n* = 354). Figure 1 shows the fitted patient-specific stress-strain curves for all patients alongside the group-mean curve.

Additional FE simulations were performed on 4 selected ATAA geometries: one representing the stiffest wall material (S1), with the other three corresponding to the least stiff wall material (C1), the thinnest wall (C5), and the thickest wall (C4), among all the simulated models. Here stress-strain curves are numbered according to their wall material properties, with ‘C’ and ‘S’ referring to more compliant and stiffer wall material properties, respectively, compared to the group-mean value, followed by a number which reflects its deviation from the group-mean curve (e.g. C1 and S1 deviate the most from the group-mean stress-strain curve). Using these 4 ATAA geometries, literature-based material properties obtained from a separate study were firstly applied for FE simulations [17]. Then, the group-mean material property fitted with the second order Ogden hyperelastic material model was also applied to understand the effect of employing different material models. The strain energy function for the Ogden model is:

$$\:W=\:\sum\:_{1}^{2}\frac{{\mu\:}_{i}}{{\alpha\:}_{i}}\:({{\lambda\:}_{1}}^{{\alpha\:}_{i}}+{{\lambda\:}_{2}}^{{\alpha\:}_{i}}+{{\lambda\:}_{3}}^{{\alpha\:}_{i}}-3)$$

where $\:{\mu\:}_{i}$ and $\:{\alpha\:}_{i}$ are empirically derived material constants, with $\:i$ = 1, 2, for the second order model, and $\:{\lambda\:}_{1}$, $\:{\lambda\:}_{2}$ and $\:{\lambda\:}_{3}$ are the principal stretches. All the fitted material constants are reported in Table 2.

Using the same 4 ATAA geometries, the influence of non-patient-specific wall thickness was examined by adopting the group-mean wall thickness of 2.1 mm (averaged from 354 samples) and a literature-based value of 1.75 mm [9–11].

**Table 2:** Material parameters: patient-specific, group- and literature-based.

Patient	C1 (kPa)		C2 (kPa)		C3 (kPa)	
C1	10.3		4.9		6.6	
C2	11.0		4.4		10.7	
C3	8.0		5.2		16.3	
C4	10.4		7.4		17.8	
C5	16.6		8.0		17.0	
C6	18.4		10.5		40.3	
C7	22.7		12.7		36.1	
C8	16.0		11.9		48.5	
S1	22.2		145.0		724.0	
S2	25.8		35.3		204.0	
S3	24.2		24.5		151.0	
S4	21.4		16.2		83.9	
S5	12.6		17.5		90.9	
Group-mean material property	17.6		17.0		74.1	
Literature-based material property (Vorp et al., [17])	82.9		259.0		28.9	
Group-mean material property (Ogden 2nd Order)	$\:{\mu\:}_{1}$ (kPa)	$\:{\alpha\:}_{1}$		$\:{\mu\:}_{2}$ (kPa)		$\:{\alpha\:}_{2}$
	9.4	12.7		23.7		3.01

### Prestress of the ATAA Model

The CTA images of all ATAA patients were obtained at diastole and thus the reconstructed geometries represented the aorta configurations under a diastolic intraluminal blood pressure, necessitating the estimation of prestress to account for physiological initial loading state. Prestress was calculated based on the method described by Votta et al. [18] and modified by Caimi et al. [19]. In brief, patient-specific brachial pressures (Table 1) were converted into central blood pressures [20], and this was followed by applying the diastolic pressure ($\:{P}_{dias}$) to the internal wall surface. The resulted Cauchy stress tensor was then imported and defined as the initial stress state for the next simulation. To avoid unrealistic deformation resulted from directly applying the full $\:{P}_{dias}$, it was ramped up in 10 increments with $\:\varDelta\:P={P}_{dias}/10$. The procedure was repeated for each $\:\varDelta\:P$ until the maximum deformation was less than 0.5 mm (the minimum pixel resolution of all CTA images). Consequently, the prestress tensor equivalent to the diastolic phase was obtained and applied in the subsequent FE simulations.

### FE Simulation and data Analysis

Prestress and FE simulations were performed using Abaqus 2019 (Dassault Systèmes, France). Both ends of the ATAA wall models were fixed in space. The calculated prestress was prescribed, and the internal pressure was ramped up from diastolic pressure to systolic pressure over 500 ms duration. The peak wall stresses calculated under systolic pressures were compared for simulations using patient-specific, group-mean and literature-based data. It should be noted that the ‘peak wall stress’ here refers to the 99th percentile maximum principal stress. The choice of using 99th percentile wall stress was made to avoid any spuriously high stress values at isolated spots.

### Statistical Analysis

The Bland-Altman method was employed to allow more detailed analysis of the similarities and differences in predicted wall stress with different choices of material properties. Statistical analyses were performed using SPSS 26.0 (IBM, Armonk, United States).

## Results

All patients have tricuspid aortic valves, whereas the clinical presentations and received treatments at the time of CTA imaging are summarized in Table 1.

### Comparisons of Simulation Results Obtained with Patient-Specific and Non-Patient-Specific Material Properties

**Fig. 2:**
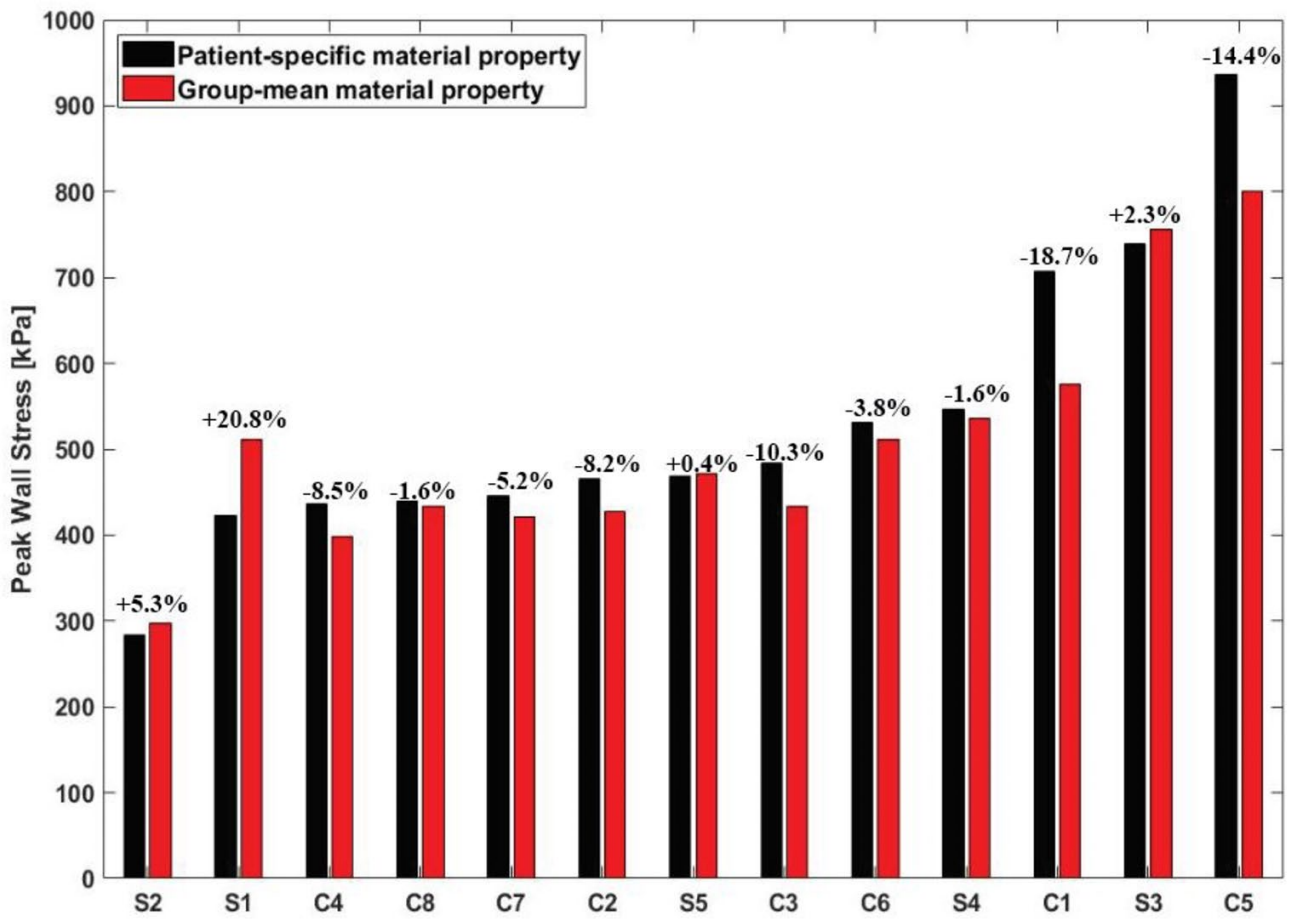
Quantitative comparison of peak wall stress, represented by the 99th percentile value, predicted by patient-specific and group-mean material properties for all simulated patients

Comparisons of peak wall stress predicted by applying patient-specific and group-mean material properties show a good overall agreement, with a maximum difference of 20.8% observed among all 13 patients (Fig. 2). Notably, the largest difference was found in S1 with the stiffest wall, where adopting the group-mean data overestimated the peak wall stress by 20.8%. In contrast, C1 representing the case with the most compliant wall led to an underestimation of wall stress by 18.7%. In general, the closer the patient-specific stress-strain curve aligns with the group-mean data (Fig. 1), the better agreement can be achieved (e.g. C8 and S6). An exception was noted in C5 (14.4%) compared to C4 (8.5%), which might be attributed to a significantly thinner wall (1.6 mm vs. 2.4 mm), and the inclusion of the aortic root in the geometric model.

**Fig. 3:**
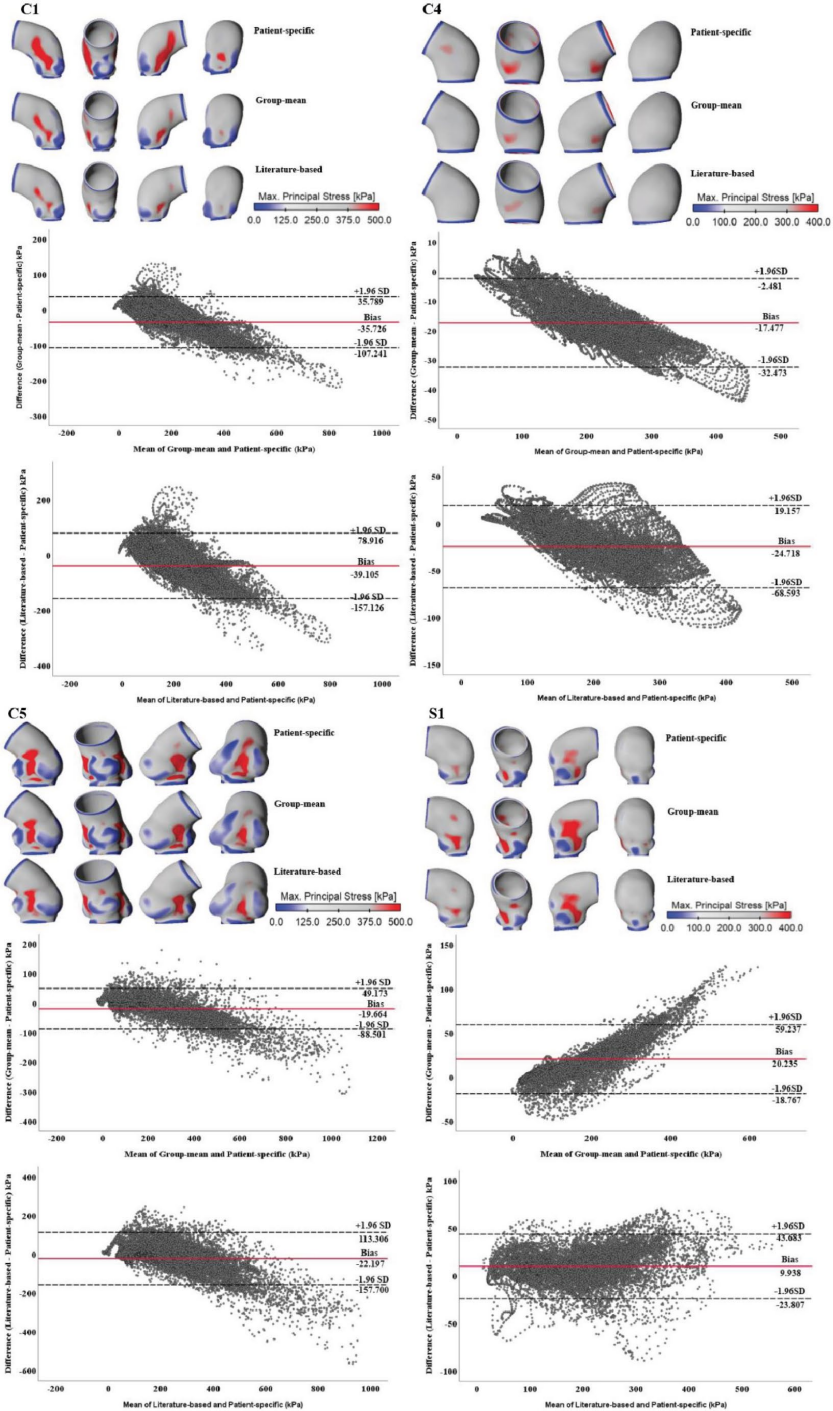
Wall stress distributions obtained from patient-specific, group-mean and literature-based material properties were compared for four representative patients. The corresponding Bland-Altman plots were placed below stress maps to show the point-by-point differences between patient-specific and group-mean material properties, as well as patient-specific and literature-based material properties

Figure 3 displays a qualitative and quantitative comparison of wall stress distributions obtained from patient-specific, group-mean and literature-based material properties, among four representative FE models. The stress distributions were qualitatively similar among these three sets of material properties, with elevated stress being observed in regions of high curvature, especially along the inner curvature. Moreover, FE models that include the aortic root (C1, C5, and S1) presented with stress concentrations between each pair of sinuses. Quantitatively, the Bland-Altman analyses revealed obvious discrepancies across different regions of the aorta, especially in areas of high wall stress. On average, predicted stress values with the group-mean material property were lower by 35.7 kPa, 17.5 kPa, and 19.7 kPa compared to those obtained with patient-specific material properties for C1, C4, and C5, respectively. Using the literature-based property produced even lower stresses, which were on average 39.1 kPa, 24.7 kPa, and 22.2 kPa lower for C1, C4, and C5, respectively. In contrast, for S1, adopting the group-mean and literature-based properties resulted in higher mean stress values by 20.2 kPa and 9.9 kPa, respectively, compared to the patient-specific model. Both ends of the ATAA wall models, where stress was low as a result of fixed support boundary condition, were excluded from the Bland-Altman analyses. Further details of the Bland-Altman analysis results can be found in Table S2 (Supplementary Materials S2).

**Fig. 4:**
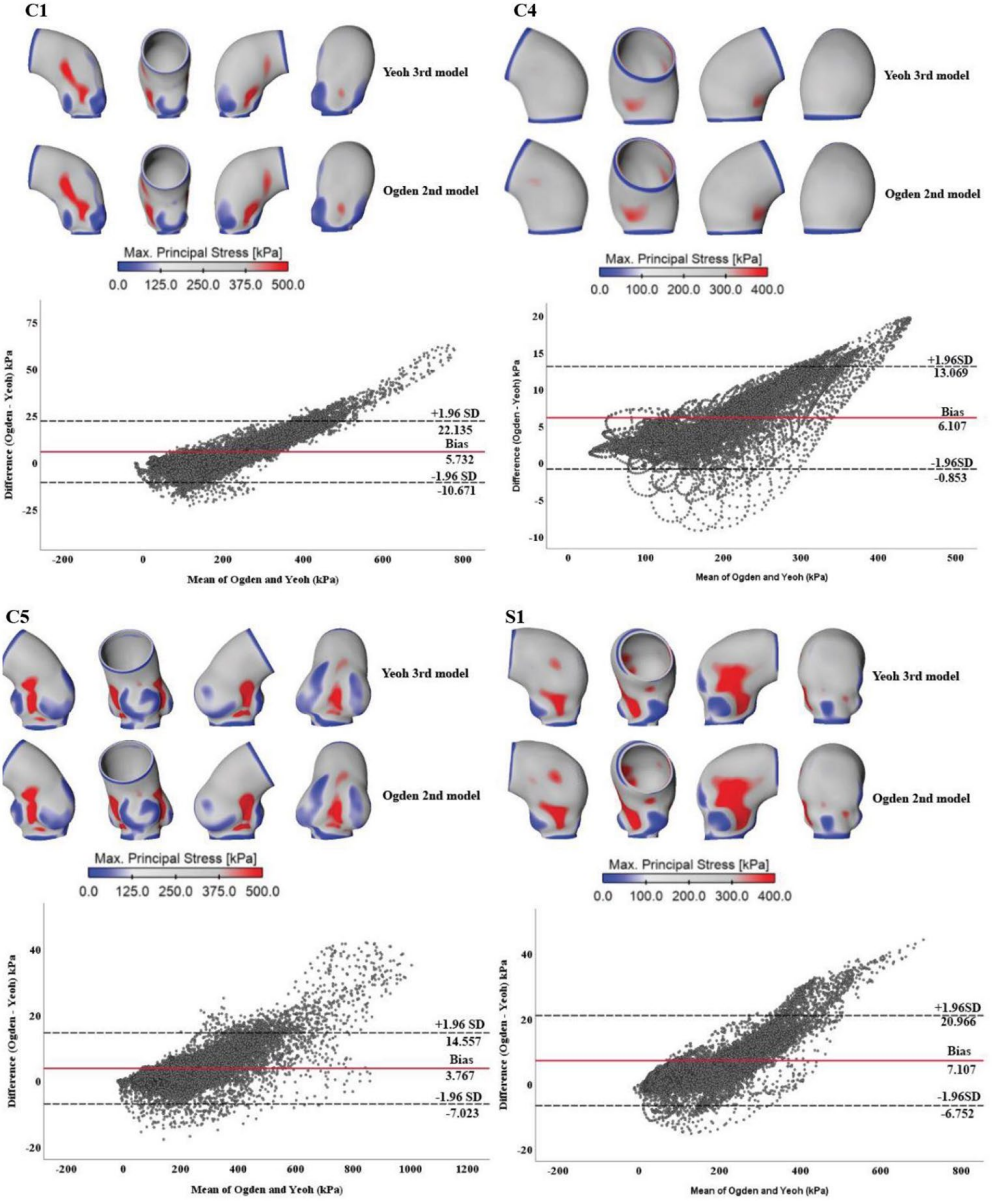
Qualitative comparison of wall stress distributions obtained from group-mean material properties fitted with two different hyperelastic material models, namely, the Yeoh third order and the Ogden second order models. The corresponding Bland-Altman plots are placed below stress maps to show point-by-point differences between the two material models

The impact of different hyperelastic material models on predicted stress distributions is presented in Fig. 4, showing negligible difference. Quantitively, the mean differences in stress values between the two material models were 5.7 kPa, 6.1 kPa, 3.8 kPa, and 7.1 kPa, forC1, C4, C5 and S1, respectively. These differences were significantly lower than the variance resulting from the use of different material properties.

**Fig. 5:**
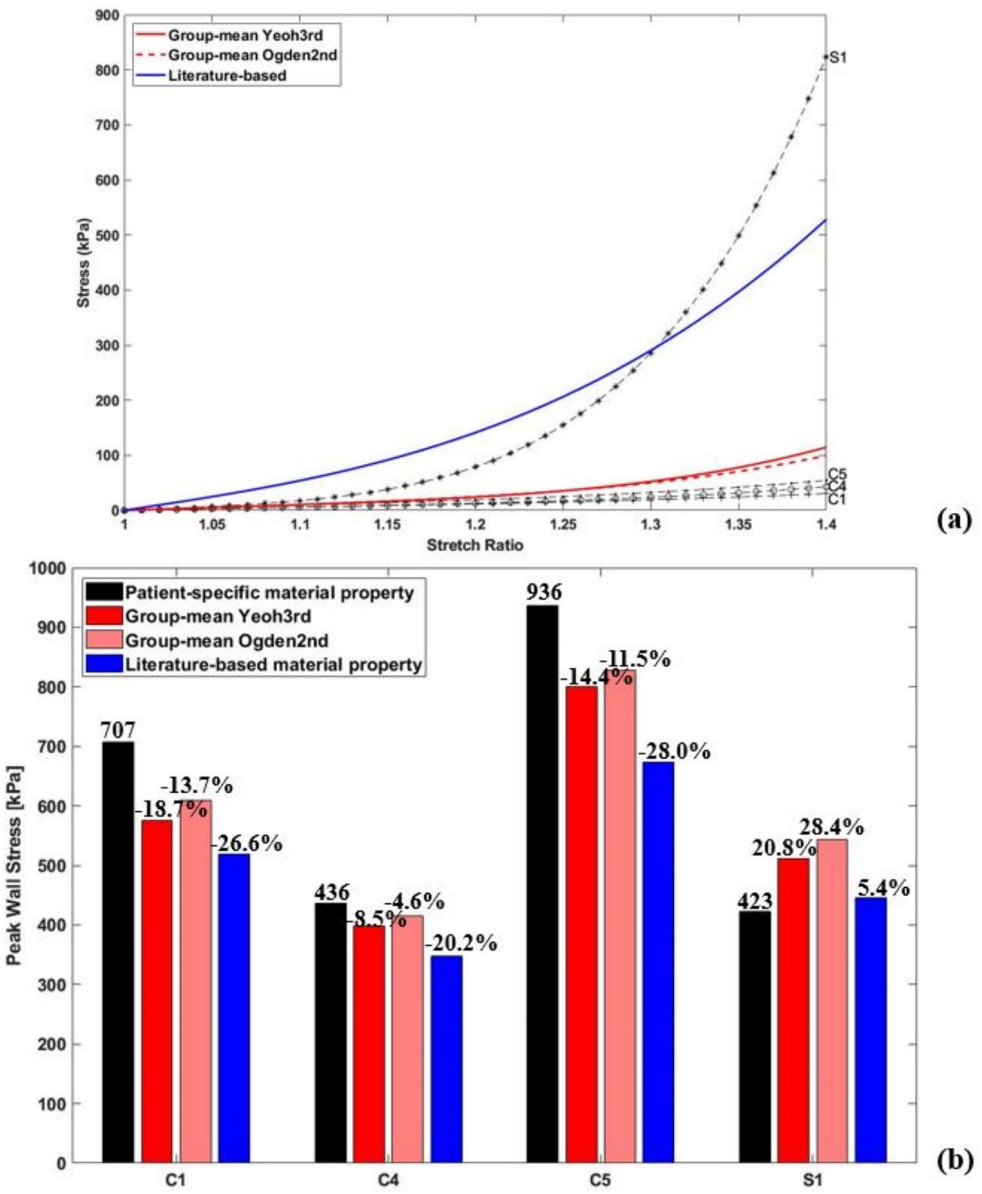
(**a**) Illustration of 4 patient-specific stress-strain curves (C1, C4, C5 and S1), two group-mean stress-strain curves fitted with Yeoh third order and the Ogden second order hyperelastic models, and a literature-based stress-strain curve. (**b**) Quantitative comparison of peak wall stress, represented by the 99th percentile value, predicted by patient-specific, group-mean and literature-based material properties for 4 representative patients

Quantitative comparisons of peak wall stress were made between the patient-specific, group-mean and literature-based material models, and the results are illustrated in Fig. 5 (b), and the corresponding representative stress-strain curves are displayed in Fig. 5 (a). Applying literature-based data caused a significantly higher reduction in predicted peak stress values (26.6%, 20.2% and 28.0%, respectively for C1, C4, and C5) compared to group-mean data (18.7%, 8.5%, and 14.4%). In contrast, for S1, whose wall property was closer to the literature-based data, thus employing group-mean data resulted in a greater increase in predicted peak stress (20.8% vs. 5.4%). Additionally, the choice of hyperelastic material model (3rd order Yeoh model vs. 2nd order Ogden model) hardly affected the stress magnitudes, with the maximum difference being 6.2%.

### Comparisons of Simulation Results Obtained with Patient-Specific and Non-Patient-Specific Wall Thickness

**Fig. 6:**
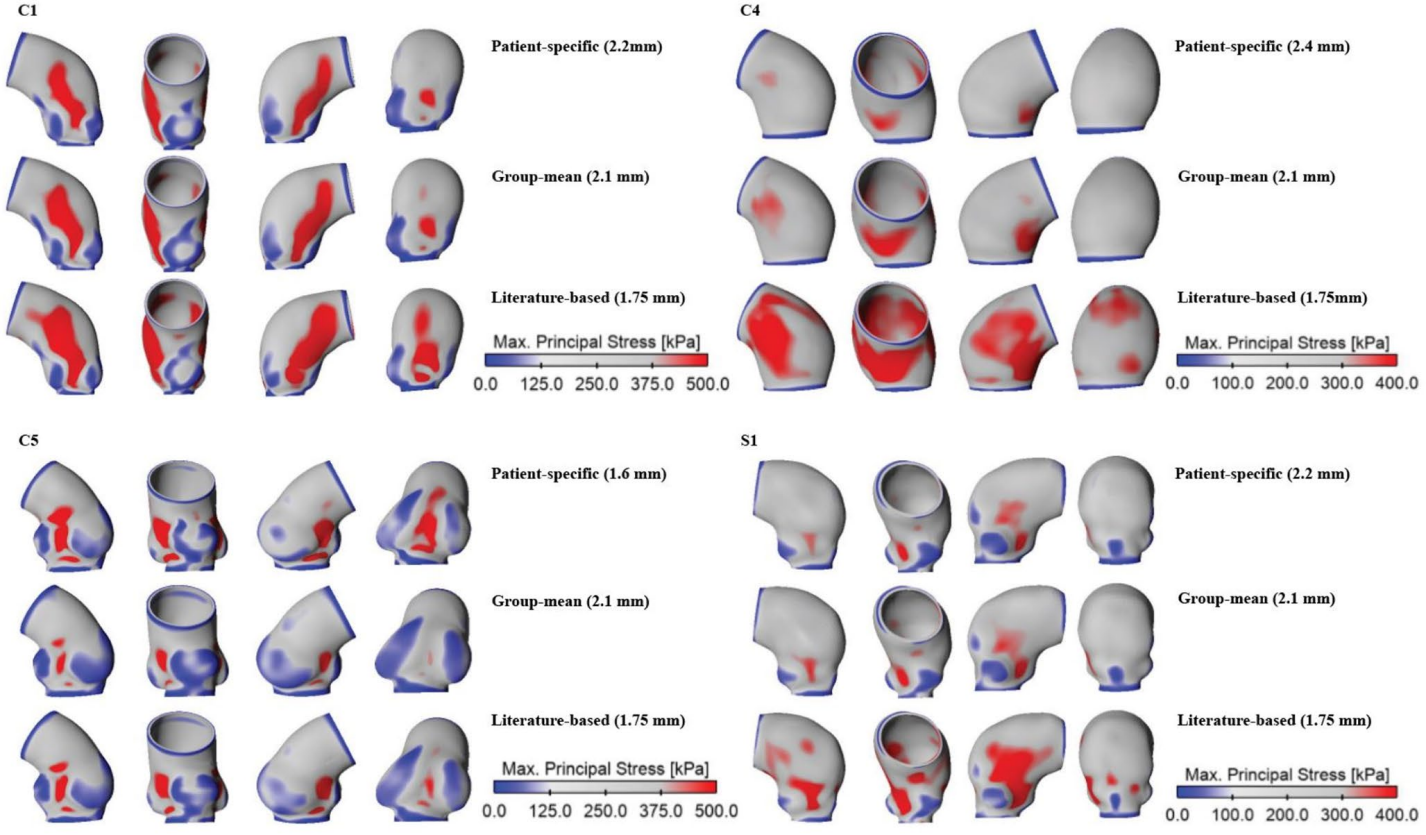
Wall stress distributions obtained from patient-specific, group-mean and literature-based wall thickness were compared for four representative patients

**Fig. 7:**
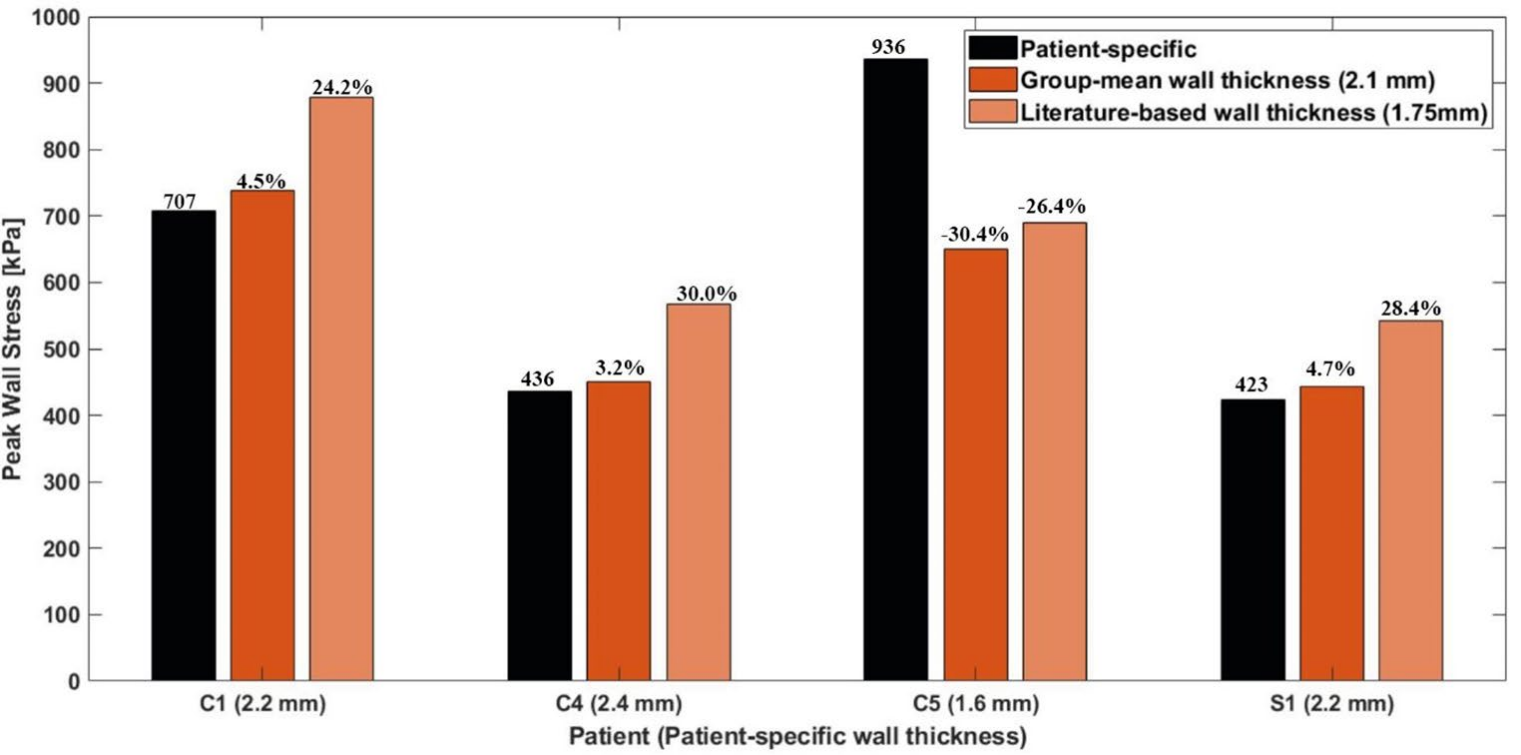
Quantitative comparison of peak wall stress, represented by the 99th percentile value, predicted by patient-specific, group-mean and literature-based wall thickness for 4 representative patients

A qualitative comparison of wall stress distributions predicted with different wall thickness values is shown in Fig. 6, featuring the same four representative models. Elevated stress also occurred in curvature regions, but the literature-based wall thickness produced greater regions of stress concentrations. Furthermore, stress concentration reduced as the aortic wall thickened. Not surprisingly, using a wall thickness close to the patient-specific value led to better agreement. Quantitatively (Fig. 7), peak stress values predicted by the group-mean data closely aligned with the patient-specific values except for C5, where using the group-mean data led to a difference of 30.4% due to the very thin wall (1.6 mm) in this case. Using a literature-based wall thickness caused substantial differences, ranging from 24.2% to 30.0%.

## Discussion

Peak wall stress was found to be significantly higher in patients with ruptured aneurysms than in non-ruptured ones [21]. Another study demonstrated that peak wall stress has a stronger correlation with aneurysm growth rate than maximal aortic diameter [22]. While these studies suggest that evaluating wall stress may provide superior predictive value for the rupture risk of aortic aneurysms compared to relying solely on diameter measurements, it is important to understand uncertainties associated with the use of non-patient-specific material properties and wall thickness. Despite great efforts have been made to determine ATAA material properties experimentally [12, 16, 23] or through an inverse method based on multi-phase CTA images [24], current biomechanical studies have used population-averaged or literature-based material properties due to challenges in either acquiring sufficient tissue samples for a specific patient group or obtaining and deriving material properties from dynamic CTA images, especially for large cohort studies [9–11, 13, 15]. Therefore, we conducted FEA to comprehensively examine the influence of employing non-patient-specific material properties and wall thickness on predicted wall stress.

The impact of material property selection on predicted wall stresses has been previously reported for abdominal aortic aneurysms (AAA) and ATAA, yielding contradictory conclusions. Polzer et al. conducted a study comparing AAA wall stresses calculated from patient-specific material properties to those derived from population-averaged material properties, demonstrating a significant influence on calculated wall stresses based on the material properties used [25]. In contrast, a study by Wang et al. suggested that using patient-specific material properties had a minor influence on either peak or averaged wall stress compared to the use of group-mean or literature-based material properties [26]. In the present study, the utilization of literature-based material properties also resulted in significant discrepancies in peak wall stress predictions: 20.2%, 5.4%, 28.0%, and 26.6%, respectively, for the four selected patients. Notably, S1 exhibited a minor difference, as the material properties for this patient were closer to the adopted literature-based values [17]. The substantial variations in the predicted peak wall stresses for the other three patients can be elucidated by Fig. 5 (a), where the stress-strain data used in the current study indicate a transition point from compliant to stiff mechanical properties at significantly higher strains compared to the literature-based data. Furthermore, stiffness at high strain is considerably lower in our data than the reported literature-based values.

Although the average mechanical property of ATAA reported by Vorp et al. [17] has been widely adopted, it is considerably stiffer than our group-mean data. This difference may arise from multiple factors, including demographic differences, disease progression, and the number, location and shape of tissue samples. For example, the mean age at surgery was 62$\:\pm\:$12.5 years in our study, compared to a mean age of 66 ± 2 years in the patient group in [17]. Additionally, a total of 354 tissue samples were included in the current study to calculate the group-mean material property, compared to 40 tissue samples in [17]. The average wall thickness was also higher in the present study with larger regional variations being observed [16]. The tensile tests were performed on dogbone-shaped samples (20 mm × 5 mm) with a 44-N load cell as described in [16], compared to rectangular samples (30 mm × 8 mm) being tested with a 25-lb (approximately 111-N) load cell, as described in [17].

Our study differs from that conducted by Wang et al. [26] in that they used an anisotropic material model. While the arterial wall is inherently anisotropic, this characteristic is largely lost when an aneurysm develops [27, 28]. The use of isotropic material properties in the FEA simulations could oversimplify the complex biomechanical behavior of the aorta which is known to be anisotropic [29–31], potentially affecting the accuracy of stress prediction. Nevertheless, implementing an anisotropic model requires detailed information on the orientation of collagen fibers throughout the aneurysmal wall. Acquiring fiber direction data typically involves advanced imaging techniques and histological analysis that were absent in our study. Consequently, we have focused solely on isotropic constitutive formulations, leveraging the existing experimental data effectively to ensure the robustness of our analysis.

The use of group-mean material properties resulted in good overall agreements in predicted peak wall stresses, with exceptions in specific cases such as S1 and C1, corresponding to the stiffest and most compliant walls in our study. Therefore, the group-mean material property will be used in our future studies of new ATAA patients when patient-specific mechanical properties are unavailable. Moreover, the group mean data could be fitted into any established hyperelastic material models, given the relatively low sensitivity of the predicted wall stresses to the choice of material models. The average difference in peak wall stresses predicted by the Yeoh and Ogden models was approximately 5%, consistent with findings reported in [14].

Using a literature-based wall thickness had a more dramatic impact on predicted wall stress, both qualitatively and quantitatively (Figs. 6 and 7), than adopting a literature-based material property. Compared to the patient-specific wall thickness, the literature-based value corresponded to a decrease in thickness by 27.1% and 20.5%, respectively for C4, and S1 and C1. As a result, the peak wall stress increased by 30.0%, 28.4%, and 24.2%, respectively. These results are comparable to another study [21], in which a 20% decrease/increase in peak wall stress was achieved by increasing/decreasing wall thickness by 25%. It should be noted that for C5, a relatively small increase in wall thickness from 1.6 mm to 1.75 mm (a 9.4% change) led to a disproportional decrease in peak wall stress from 936 kPa to 689 kPa (a 26.4% decrease). As a result, the highest peak wall stress was found in C1 when using group-mean or literature-based wall thickness, even though C5 had the highest peak wall stress with patient-specific wall thickness. This indicates that ATAA models with a thinner aortic wall are more sensitive to the choice of wall thickness for FEA: a small change in wall thickness could result in similar impact on wall stress predictions as a larger alteration in wall thickness. Despite the Law of Laplace stating that mean stress is inversely proportional to thickness in a cylinder, it exhibits nonlinear behaviour in complex geometries, with the exact degree of sensitivity determined by the radius. The geometric model of C5 is different compared to other models, as this model has a shorter portion of the ascending aorta but significantly larger sinuses. For the other three simulated patients, employing a group-mean wall thickness produced good wall stress predictions, with a difference lower than 5% compared to the patient-specific simulations.

**Fig. 8:**
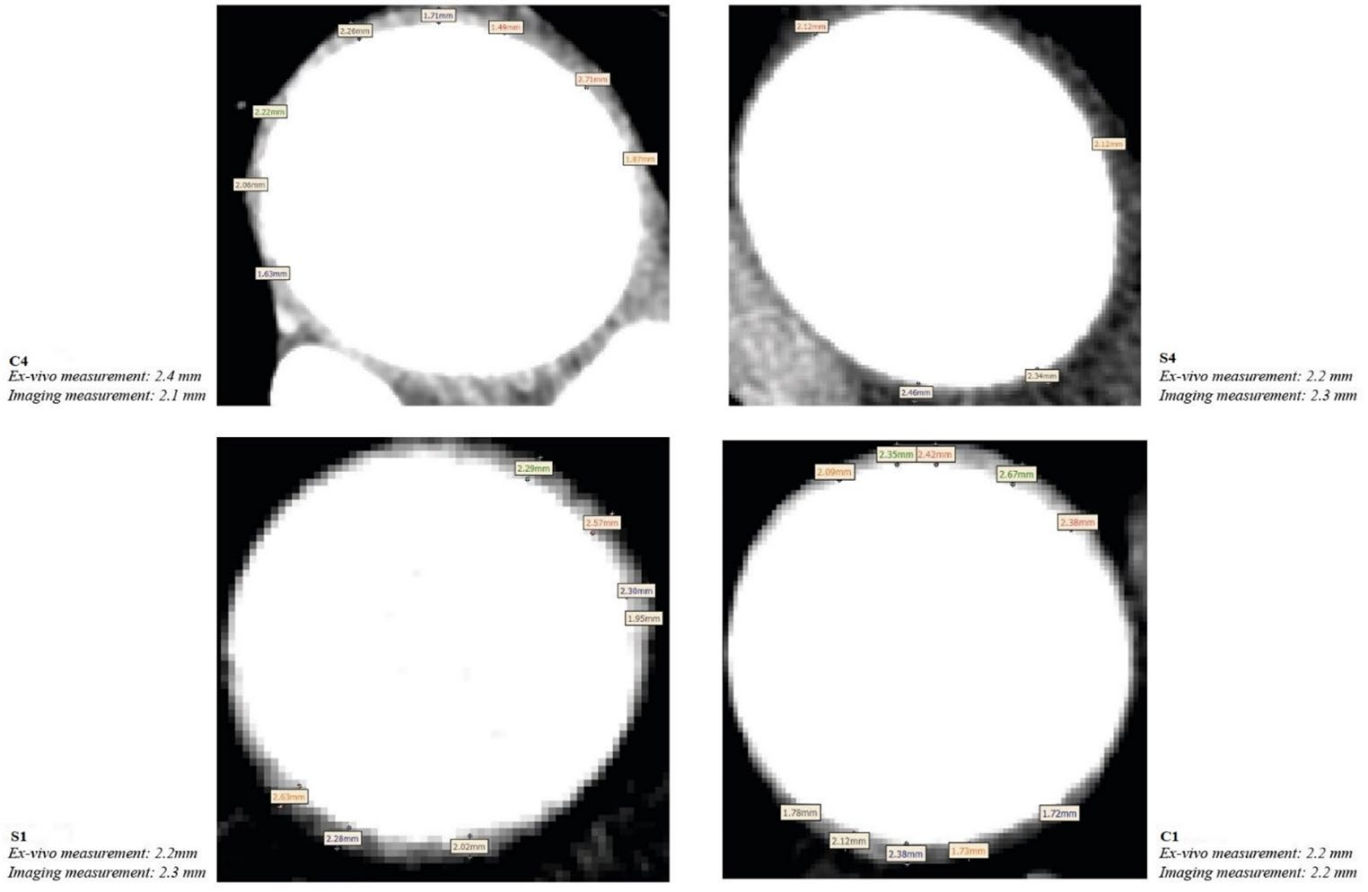
Computed tomography angiography images with a cross-sectional slice showing wall boundaries in 4 selected patients. Wall thickness measurements taken from imaging data were found to be comparable to those obtained ex-vivo

While no reliable non-invasive method is available for in-vivo measurement of aortic wall thickness, current FEA studies often rely on ex-vivo measurements [7, 8] or data reported in the literature. However, in cases where ex-vivo tissues are not available, it may be worth exploring the available imaging data to identify cross-sectional slices with clearly depicted wall boundaries for thickness estimations. Fig. 8 gives an example of CTA images of 4 patients where wall boundaries can be identified clearly. The measured wall thicknesses from these cross-sectional slices were comparable to the ex-vivo measurements, with errors ranging from 0 to 0.3 mm. Moreover, the averaged wall thickness based on these 4 patients was 2.25 mm, closely matching the group-mean wall thickness. Therefore, this approach could be considered for patient-specific wall stress predictions instead of applying literature-based wall thickness. On the other hand, wall thickness measured in-vivo is expected to be lower than ex-vivo measurement [24, 32], resulting from radial contraction induced by the Poisson effect when the tissue is loaded [32]. The slightly smaller or equal *ex-vivo* thickness compared to *in-vivo* wall thickness in our study (Fig. 8) might be attributed to human errors that were inevitable.

The peak wall stresses predicted based on patient-specific data varied between 280 kPa and 940 kPa (Fig. 1), which were well below the experimentally measured mean rupture stress of 1280 kPa, as reported by Trabelsi et al. [8]. However, individual rupture stress exhibited significant variability, ranging from 760 kPa to 2330 kPa [8]. Despite the potential for improved rupture risk prediction based on patient-specific rupture stress, such information was not available in our study. On the other hand, the averaged ultimate tensile strength (UTS) was reported to be 690 kPa [16], with circumferential UTS (mean: 850 kPa) being much higher compared to longitudinal UTS (mean: 530 kPa). The peak wall stress was located along the inner curvature of all ATAA models, irrespective of the material properties or wall thickness. Despite the relatively small mean deviations in predicted stress between patient-specific and group-mean wall properties or literature-based values, the Bland-Altman plots revealed significant differences in regions of either low or high wall stresses. Since our focus is on areas of high wall stress, using literature-based material properties caused maximum differences of 354 kPa, 145 kPa, 674 kPa, and 100 kPa, for C1, C4, C5, and S1, respectively, in these regions. Particularly for C5, the rupture risk would be significantly underestimated using the literature-based material property with wall stress being underestimated by 674 kPa, which is almost half of the reported mean rupture stress [8]. Although using the group-mean material property improved stress predictions, the maximum deviation could still be as large as 227 kPa and 197 kPa for C1 and C5, thereby underestimating the risk of rupture.

This study provides a detailed quantitative assessment of the errors introduced by using non-patient-specific wall material properties and thicknesses, a topic that has been inadequately explored in existing literature. In addition, Bland-Altman plots were employed to quantify discrepancies in wall stress predictions across models with different material properties. Our results highlight the importance of employing patient-specific material properties and wall thickness for accurate prediction of wall stress in ATAA, especially when the predicted stress is used to assess the risk of aneurysm rupture or in surgical planning for individual patients. However, it is currently impossible to obtain such information in vivo, underscoring the need for developing non-invasive methods to determine these patient-specific data. Given the challenges in obtaining patient-specific data non-invasively, the development of artificial intelligence-based predictive tools may reduce the reliance on biomechanical parameters by incorporating clinical and morphological indices. Such models can potentially predict patient outcomes with reduced dependence on FE model accuracy [33].

### Limitations

The major limitation of the present study was the assumption of uniform wall thickness, despite significant regional variations in aortic thickness having been demonstrated in pathological specimens [16]. Incorporating local wall thickness has been reported to significantly increase peak wall stress magnitudes and alter their regional distributions in previous FEA studies of both AAA and TAA models [34, 35]. However, as aforementioned, regional variation in wall thickness could not be identified from the CTA images, leading to the common practice of assuming uniform wall thickness [7–15]. Second, although the experimentally derived material parameters of the ATAA wall may differ from the in vivo condition, there is currently no methodology to in-vivo estimate material behaviour, making experimental material parameters the most accurate descriptors for ATAA material properties. In addition, FE simulations were based on isotropic material constitutive formulations, and the impact of anisotropic material models [29–31] would be detected if the information on fibre directions could be obtained. Given the challenges in obtaining patient-specific data for FE simulations, development of artificial intelligence-based prediction models may diminish the reliance on biomechanical parameters by incorporating clinical and morphological indices. Such models enable the prediction of patient outcomes with reduced dependence on FE model accuracy [33]. Additionally, the aortic root motion was neglected, whereas previous FE studies have revealed that the aortic root downward motion could significantly increase the longitudinal stress in the ascending aorta [14, 36]. Therefore, aortic root motion should be included in future FE simulations when patient-specific data become available. Finally, only 13 patients with available CTA images were included for FE simulations. However, the number of patients, especially those whose tissue samples underwent mechanical tests is larger than other similar studies [7, 18, 26].

## Conclusion

Our study highlights the significant impact of using non-patient-specific material properties and wall thickness in FE simulations on predicted peak wall stresses in ATAA patients, resulting in a maximum discrepancy of 28.0% and 30.4%, respectively. Moreover, the Bland-Altman plots showed that rupture risk would be significantly underestimated using the literature-based material property with wall stress being underestimated by 674 kPa in one patient, which is almost half of the reported mean rupture stress [8]. Hence, patient-specific data should be used whenever possible for more reliable risk stratification. Recognising the substantial influence of wall thickness on predicted wall stress, especially in patients with thinner aortic walls, we propose exploring the possibility of thickness measurements from imaging data when ex-vivo tissues are unavailable. Even information from a single slice may enable FE models to incorporate more accurate patient-specific wall thickness. Future research efforts should focus on developing techniques for non-invasive determination of patient-specific wall material parameters and wall thickness, while also incorporating regional variations in wall thickness to enhance the reliability of biomechanical models. This will allow a more precise and personalized approach for predicting the rupture risk of ATAAs, which can significantly impact clinical decision-making and improve patient outcomes.

## Electronic Supplementary Material

Below is the link to the electronic supplementary material.

Supplementary Material 1
