# Supplementary material for: The Influence of Material Properties and Wall Thickness on Predicted Wall Stress in Ascending Aortic Aneurysms: A Finite Element Study
Source: Cardiovasc Eng Technol. 2024 Oct 25;16(1):52–65. doi: 10.1007/s13239-024-00756-9 (PMC11821800; doi:10.1007/s13239-024-00756-9)
Supplement: Supplementary file 1 — Supplementary Material 1 [file 13239_2024_756_MOESM1_ESM.docx]

**Supplementary Materials**

**The Influence of Material Properties and Wall Thickness on Predicted Wall Stress in Ascending Aortic Aneurysms: A Finite Element Study**

**Yu Zhu^1^, Selene Pirola^2^, M. Yousuf Salmasi^3^, Sumesh Sasidharan^4^, Serena M. Fisichella^1,5^, Declan P. O’Regan^6^, James E. Moore Jr^4^, Thanos Athanasiou^3^ and Xiao Yun Xu^1^***

1. Department of Chemical Engineering, Imperial College London, London, UK
2. Department of Biomechanical Engineering, Delft University of Technology, Delft, Netherlands
3. Department of Surgery and Cancer, Imperial College London, London, UK
4. Department of Bioengineering, Imperial College London, London, UK
5. Politecnico di Milano, Milan, Italy
6. MRC Laboratory of Medical Sciences, Imperial College London, London, UK

*** Correspondence:**Professor Xiao Yun Xu [yun.xu@imperial.ac.uk](mailto:yun.xu@imperial.ac.uk)

# **S1. Mesh sensitivity tests**

Mesh sensitivity tests were performed to ensure that a mesh independent solution has been achieved. Static structural analyses were performed for different meshes and the predicted maximum principal stress values were compared. The results of mesh sensitivity tests for two representative cases are summarised in Table S1 and Figure S1. Solutions were considered mesh independent when the monitored parameters differed by less than 1% between two successively refined meshes. Consequently, M2 of both cases were deemed sufficient and chosen for the final simulations.

Table S1. Mesh sensitivity tests results showing comparison of the peak maximum principal stress between different meshes.

|  | ATAA model 1 | ATAA model 2 |
| --- | --- | --- |
|  | Peak Max. Principal Stress (MPa) | Peak Max. Principal Stress (MPa) |
| M1 | 0.1973 | 0.1519 |
| M2 | 0.2103 | 0.1609 |
| M3 | 0.2120 | 0.1615 |
| Difference in M1/M2 (%) | 6.6 | 5.9 |
| Difference in M2/M3 (%) | 0.8 | 0.4 |


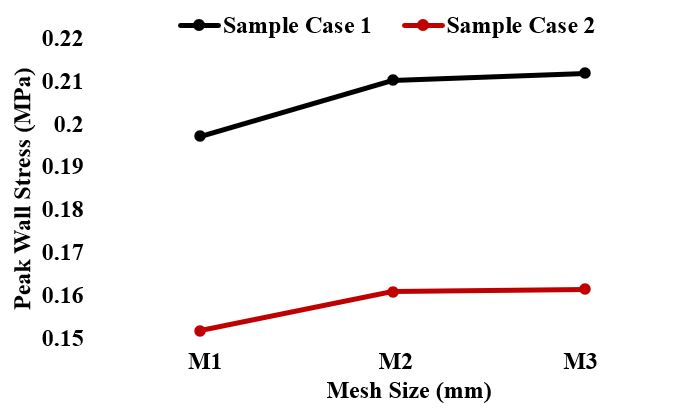


Figure S1. Plot of peak maximum principal stress versus different mesh sizes for two ATAA models.

# **S2. Bland-Altman analyses**

Table S2. Bland-Altman analyses results for 4 selected patients, among various comparisons.

|  | Bland-Altman comparison of stress (kPa) between patient-specific and group-mean material properties | | | | |
| --- | --- | --- | --- | --- | --- |
| Patient | Patient-Specific | Group Mean | Bias | 95% LOA | |
|  |  |  |  | Lower | Upper |
| C1 | 262.901±132.133 | 227.175±103.256 | -35.726 | -107.241 | 35.789 |
| C4 | 221.521±72.991 | 204.044±66.797 | -17.477 | -32.473 | -2.481 |
| C5 | 261.966±165.501 | 242.302±140.080 | -19.664 | -88.501 | 49.173 |
| S1 | 192.661±81.931 | 212.896±98.712 | 20.235 | -18.767 | 59.237 |
|  | Bland-Altman comparison of stress (kPa) between patient-specific and literature-based material properties | | | | |
| Patient | Patient-Specific | Population Mean | Bias | 95% LOA | |
|  |  |  |  | Lower | Upper |
| C1 | 262.901±132.133 | 223.796±90.138 | -39.105 | -157.126 | 78.916 |
| C4 | 221.521±72.991 | 196.803±58.996 | -24.718 | -68.593 | 19.157 |
| C5 | 261.966±165.501 | 239.769±121.263 | -22.197 | -157.700 | 113.306 |
| S1 | 192.661±81.931 | 202.598±85.562 | 9.938 | -23.807 | 43.683 |
|  | Bland-Altman comparison of stress (kPa) between Yeoh 3^rd^ order and Ogden 2^nd^ Order material models | | | | |
| Patient | Yeoh 3^rd^ order | Ogden 2^nd^ order | Bias | 95% LOA | |
|  |  |  |  | Lower | Upper |
| C1 | 227.175±103.256 | 232.907±110.524 | 5.732 | -10.671 | 22.135 |
| C4 | 204.044±66.797 | 210.151±69.594 | 6.107 | -0.853 | 13.067 |
| C5 | 242.302±140.080 | 246.069±144.256 | 3.767 | -7.023 | 14.557 |
| S1 | 212.896±98.712 | 220.003±104.618 | 7.107 | -6.752 | 20.966 |
